# Distinct ageing- and androgen-dependent effects on cGMP signalling proteins in three male rat urogenital organs (bladder, prostate, epididymis)

**DOI:** 10.1186/1471-2210-11-S1-P51

**Published:** 2011-08-01

**Authors:** Dieter Müller, Michail S Davidoff, Ralf Middendorff

**Affiliations:** 1 Institute of Anatomy and Cell Biology, University of Giessen, Germany; 2 Institute of Clinical, Chemistry, University of Hamburg, Germany

## Background

Aging of the male reproductive system leads to changes in endocrine signalling and is frequently associated with the emergence of bothersome conditions owing to prostate hyperplasia and bladder dysfunctions. Recent findings suggest that urogenital organs are promising targets for therapeutic interventions with inhibitors of the cGMP-degrading phosphodiesterase 5 (PDE5) [1]. However, the cGMP signalling system in these tissues and possible age-related alterations are only partially characterized. This study investigated key proteins of cGMP pathways in bladder, prostate and epididymis of young (3 months) and old (23-24 months) Wistar rats. To address androgen-dependent effects, we (i) used an experimental model, where injection of ethane dimethane sulphonate (EDS) into adult rats evokes a temporary elimination of the testosterone-producing Leydig cells [2] and (ii) examined effects induced by castration and testosterone supplementation.

## Methods and results

Western blot analyses aimed to characterize the levels of PDE5, soluble (sGC) and particulate (GC-A, GC-B) guanylyl cyclases, endothelial nitric oxide synthase (eNOS) and cGMP-dependent protein kinase I (cGKI) in membrane and cytosolic protein fractions. Data revealed a strikingly low abundance of all these proteins in prostate. In contrast, the bladder was distinguished by exceptionally high levels of PDE5, cGKI and eNOS, each resembling those in lung tissue. The epididymis was characterized by a predominance of particulate (in particular GC-B) versus soluble (sGC) guanylyl cyclases and lowest PDE5 tissue concentrations.

As evidenced also by membrane guanylyl cyclase assays, ageing did not affect the three (GC-A, GC-B, sGC) cGMP-generating enzymes in all organs. However, and supported by PDE activity studies, we recognized a pronounced age-related decrease (by nearly 50%) of PDE5 in bladder. Membrane-associated (but not cytosolic) cGKI concentrations were markedly reduced in the aged epididymis. On the other hand, prostatic cGKI increased with ageing. Androgen withdrawal during temporary Leydig cell elimination induced a massive (>12-fold) upregulation of cGKI in prostate but not other (penis, epididymis) androgen-dependent organs. Castration elicited an equally organ-selective upregulation of cGKI in prostate. Neither EDS treatment nor castration evoked increases in other kinases (PKA, Akt, PI3K, GSK-3) examined. Testosterone supplementation reduced the increased cGKI levels in prostates of castrated rats in a dose-dependent manner.

## Conclusion

The findings may have significance for ageing-associated pathologies in the male lower urinary tract and identify cGKI as an androgen-sensitive signalling protein in prostate.
